# A wide-band bio-chip for real-time optical detection of bioelectromagnetic interactions with cells

**DOI:** 10.1038/s41598-018-23301-w

**Published:** 2018-03-22

**Authors:** Caterina Merla, Micaela Liberti, Paolo Marracino, Adeline Muscat, Antoine Azan, Francesca Apollonio, Lluis M. Mir

**Affiliations:** 1 Laboratory of Vectorology and Anticancer Therapies, UMR 8203, CNRS, Univ. Paris-Sud, Gustave Roussy, Université Paris-Saclay, 114 rue E. Vaillant, 94805 Villejuif, France; 2 National Italian Agency for New Technology Energy and Sustainable Economic Development (ENEA), Division of Health Protection Technologies, via Anguillarese 301, 00123 Rome, Italy; 3 “Sapienza” University of Rome, Department of Information Engineering Electronics and Telecommunications, via Eudossiana 18, 00184 Rome, Italy

## Abstract

The analytical and numerical design, implementation, and experimental validation of a new grounded closed coplanar waveguide for wide-band electromagnetic exposures of cells and their optical detection in real-time is reported. The realized device fulfills high-quality requirements for novel bioelectromagnetic experiments, involving elevated temporal and spatial resolutions. Excellent performances in terms of matching bandwidth (less than −10 dB up to at least 3 GHz), emission (below 1 × 10^−6^ W/m^2^) and efficiency (around 1) have been obtained as revealed by both numerical simulations and experimental measurements. A low spatial electric field inhomogeneity (coefficient of variation of around 10 %) has been achieved within the cell solutions filling the polydimethylsiloxane reservoir of the conceived device. This original bio-chip based on the grounded closed coplanar waveguide concept opens new possibilities for the development of controlled experiments combining electromagnetic exposures and sophisticated imaging using optical spectroscopic techniques.

## Introduction

Nowadays, one of the main aims of bioelectromagnetic studies copes with the use of electromagnetic fields to expose biological cells or tissues under a wide range of frequencies and amplitudes and to solidly establish effects exploitable for biological, technological and medical applications.

One of the main limitations in translating electromagnetic induced effects (from DC to tens of GHz) in advanced applications, apart from the well-established phenomena at the basis of human health protection regulations^1–3^ (i.e. nerve stimulation at low frequencies and thermal effects at high frequencies), it is a lack of knowledge on defined and verified mechanisms of interaction^4^. In particular, some effects have been observed on oxidative stress, membrane channel activity, activation of signaling pathways, and epigenetic modifications. Such effects can be amplified or modulated by co-exposure factors and they also can involve adaptive phenomena^5–9^.

There is a general consensus that, to initiate or promote effects in biological systems, electromagnetic fields must give rise to a series of events that, starting with a field interaction at molecular level altering molecule charge distribution, chemical state, or energy, ultimately lead to some biological outcomes. It is likely that such transduction step takes place at the cell membrane site. Considering that such an event can be very slight, new biological observables should be investigated with new spectroscopic techniques such as for example oxidative products, and protein involvement in order to fully understand the whole chain of events at the basis of the observed effects. Indeed such effects, even if in some cases need further investigation and rigorous assessment, may have direct implications in oncology or may be considered for potential adjuvancy in anticancer therapies, neurology, controlled drug delivery, wound healing, osteogenesis, regulation of inflammation processes, stem cell based therapies and tissue engineering^10–14^.

In this context, one of the most relevant electromagnetic modalities to treat biological systems (*in vitro and in vivo*) deals with electroporation (or electropulsation) techniques. Cells electropulsation has rapidly expanded and various medical applications are available or under development. These approaches employ short duration electric pulses of high intensity (from few hundreds of V/m to tens of MV/m) to affect cells and tissues. Specific stimuli are used depending on the specific applications. For example, relatively long electric pulses (in the µs and ms range, with intensities ranging from few tens of kV/m up to few MV/m) are used to permeabilize cell membranes. These high voltage pulses are applied in electrochemoterapy^15^, electro-gene transfer^16^, calcium electroporation^17^, electrostimulation of immune system^18^, and intracellular calcium control^19^.

Shorter electric pulses (in the ns time scale, with intensities up to tens or hundreds of MV/m) can be applied to permeabilize also the cell intracellular organelles and hence manipulate intracellular functions such as calcium fluxes and various cell signaling pathways^20–24^. Medical applications of electropulsation in the nanosecond time scale appear very wide and span from potential smart drug delivery^25,26^, cancer ablation^27^, neuronal stimulations^28^ to the possible use of nano-pulses in regenerative medicine^23^.

Further, a recent research trend promotes the application of pulses of much shorter duration down to hundreds of ps. These wide-band signals allow non-invasive deep tissue applications using suitably designed antennas^29,30^.

Beside wide-band pulsed electric stimuli, some groups also demonstrated that sinusoidal electromagnetic signals applied for a very limited time (e.g. few ms) are able to induce calcium release into the intracellular environment at lower field amplitudes compared to the ones required for microsecond and nanosecond rectangular pulses^31^. This result opens the way to new types of potential applications in medicine and biology using simple sinusoidal electromagnetic fields, even if a wide lack of knowledge still exists concerning potential biological effects at variable field amplitudes and frequencies (from few kHz to few GHz) as noted in^31,32^.

For all these types of electric and electromagnetic signals, the permeabilization of the cell membrane and of the membranes of the intracellular organelles seems mediated by the formation of pores occurring above a given transmembrane potential threshold, as depicted by molecular dynamics (MD) simulations looking at a very short time scale (ns)^33–36^. A direct experimental observation of pores has not yet been achieved and only transport of molecules supports the hypothesis of pore creation and maintenance under electromagnetic field application on time scales longer than the ones theoretically predicted by MD^37,38^. Other hypotheses to support membrane electropermeabilization propose the possible role of lipid oxidation^39^. The direct involvement of voltage gated calcium channels for the observed ion transport has been also shown^40^ as well as the role of mechanical stress^22^ in permeabilization. All these events have been observed within a few minutes after the electromagnetic field delivery. Nonetheless, a concomitance of all these events and even other phenomena (e.g. cytoskeleton modifications, gap junction communication alteration) cannot be excluded^41^. Therefore, suitable (i.e. highly sensitive in time and space, and providing accurate outcomes) techniques are needed to assess the underlined hypotheses of mechanisms of interaction^42^.

Important techniques to deep into chemical modifications of the matter, as the ones potentially induced by electromagnetic fields on cell membrane, can involve the use of linear and non-linear photonics (e.g. Raman spectroscopy, second harmonic generation, coherent anti-Stokes Raman scattering) as proposed in^43–47^. Morphological and functional cell and tissue modifications can be also nicely attained with sophisticated microscopy (e.g. confocal and multiphoton) using fluorescence dyes and ultra-fast CCD cameras down to the sub-microsecond time resolution^48–50^.

In all these complex imaging techniques usable to detect the mechanisms of interaction of electromagnetic fields with the biological samples, with high time/space resolutions, a very important requisite is to analyze the cells or tissues in real-time^51–53^. It means that electric or electromagnetic exposure has to be performed during images and data acquisition, or that the planned stimulation has to be delivered immediately before the beginning of the data acquisition using precise synchronization protocols.

Therefore, a bio-chip for *in vitro* electromagnetic exposure and real-time optical spectroscopy and imaging has to respect specific prerequisites which are:

- a frequency band matching up to some GHz;
- the electromagnetic compatibility in the very close proximity of the microscope electronics and cameras, according to current guidelines;
- the planarity in order to be integrated in a microscope stage;
- the transparency of a specific region named “visibility windows” as imposed by the optical applications;
- the assurance of a good cell preservation (biocompatibility).

In this paper, the design and experimental characterization of an optimal electromagnetic structure to fulfill these requirements are presented. The structure is based on a coplanar waveguide with a ground bottom plane connected to lateral ground electrodes. We refer to this type of architecture with the term “closed”: our novel bio-chip is thus a grounded closed coplanar waveguide (GCCPW). This bio-chip was first designed on the basis of analytical formulations then analyzed using numerical simulations, in the air and in the presence of biological samples (i.e. dosimetry). The GCCPW was fabricated and characterized in the frequency and time domains to verify the fulfillment of the bandwidth adaptation, the electric field levels reached within the biological solution (efficiency), and the homogeneity and irradiative performances in terms of emitted power density. Comparisons between simulations and experimental verifications have been performed demonstrating successful results.

## Design of the GCCPW

**Figure 1:**
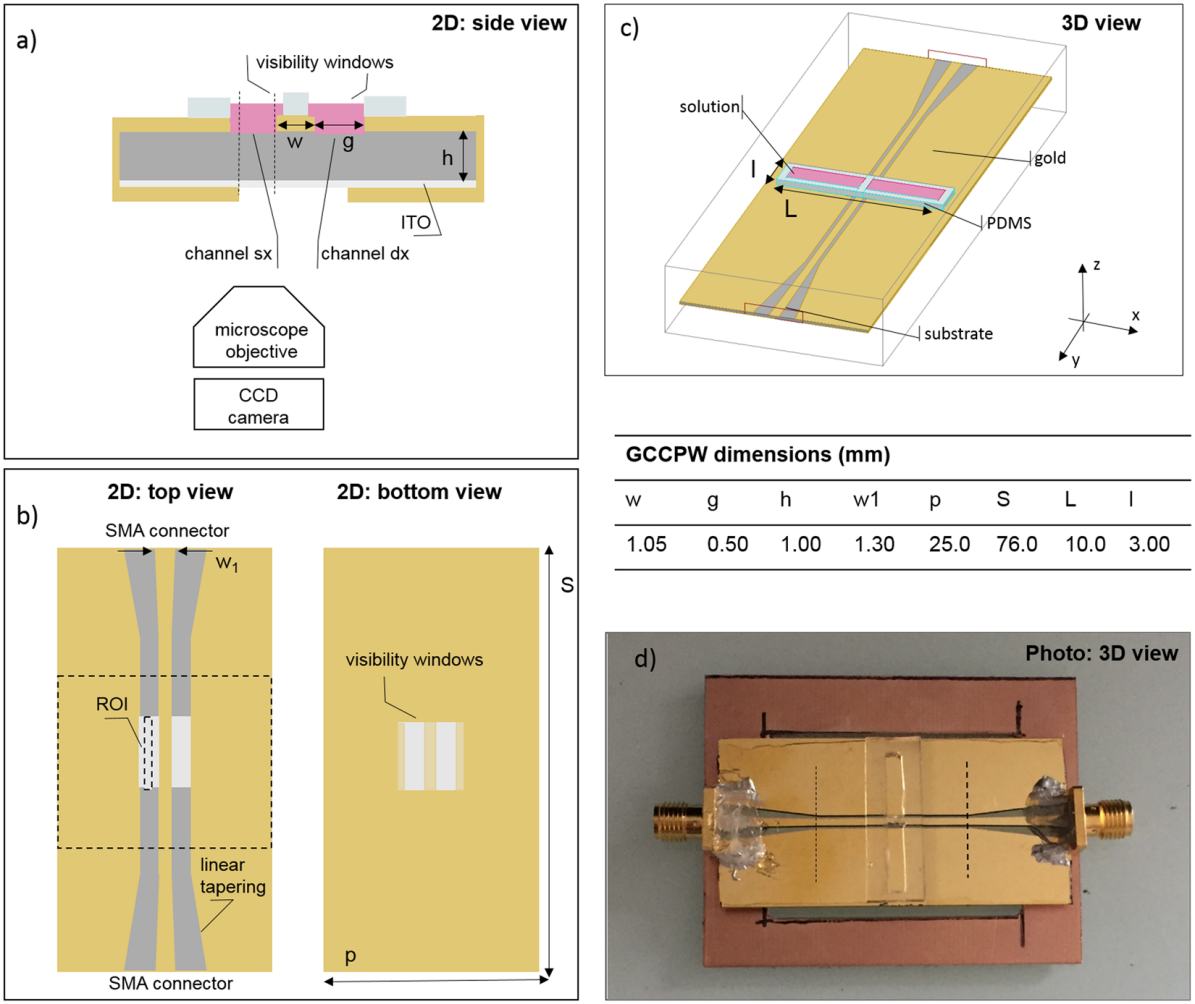
(**a**) GCCPW 2D side view: visibility windows and ITO layer are shown. (**b**) 2D top view: the linear tapering is sketched as well as the aperture on the bottom substrate side. (**c**) 3D view of the designed GCCPW. (**d**) Photo of the fabricated GCCPW with SMA connectors and a PCB support to provide mechanical stability when the GCCPW is placed in a microscope stage.

To maximally limit electromagnetic radiations in real-time applications, an original guiding structure has been conceived, based on a coplanar waveguide with a ground plane. In our design the lateral ground strips are connected to the ground plane at the bottom side of the bio-chip. This new configuration for a coplanar waveguide largely limits the electromagnetic emissions and hence it is an optimal choice for real-time experiments in the stage of a microscope. This novel device is named grounded closed coplanar waveguide “GCCPW” (see Fig. 1). To assure visibility of cells, the prerequisite for imaging techniques, the transparent calcium fluoride (CaF_2_) substrate was chosen, as it is one of the best substrates for linear and non-linear optics, guaranteeing the lowest background signals and the absence of absorption on a wide range of vibrational frequencies. A square aperture within the metallic ground plane was made to assure a continuous light path from the top of the GCCPW to the bottom ground plane down to the microscope objective (visibility windows shown in Fig. 1a). To assure the electrical continuity of the ground plane, needed to maximally reduce the electromagnetic radiation near the microscope objective, a conductive (and transparent) indium thin oxide (ITO) layer covers the visibility windows, which maintains at the same time the required light path transparency for imaging and data acquisition by the CCD camera (Fig. 1a).

To allow the focusing of lasers needed in linear and non-linear optics experiments, the coplanar waveguide strips have to be separated of at least few hundreds of micrometers. We chose an inter-electrode distance of 0.5 mm (‘g’ in Fig. 1, panel a) to allow easy laser passage (the laser beam diameter at the focus plan can be in the order of one hundred microns).

Using formulas given in^54^, we defined the width of the central electrode (‘w’ in Fig. 1, panel a) as a function of the substrate permittivity (ε_r_ = 2.8), as well as the electrode gap distance and the substrate thickness (all dimensions are reported in Fig. 1) to get a 50 Ω matching on a large band.

Since in our bio-chip lasers may have to reach the cells also from the top of the cell containing volume, a double open biocompatible polydimethylsiloxane (PDMS) reservoir for cells was designed (Fig. 1c).

## Results

### Unloaded GCCPW analysis in the frequency domain: S parameters and emitted power density

**Figure 2:**
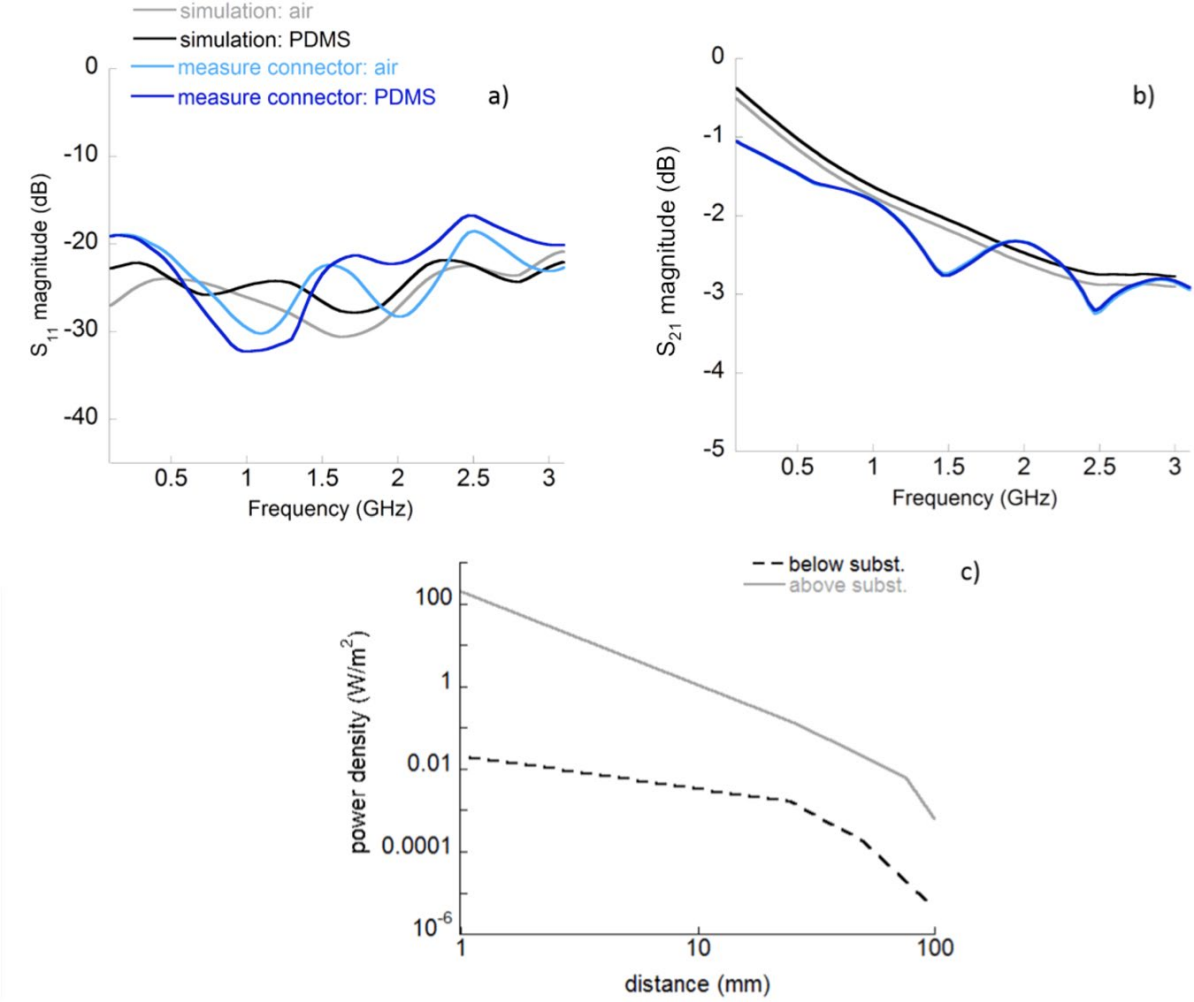
(**a**) S_11_ of the GCCPW in air, with and without the PDMS reservoir, from simulations and measurements. (**b**) The corresponding S_21_ in air, with and without the PDMS reservoir. (**c**) Emitted power densities of the GCCPW in air (for an input voltage of 1 V).

The simulated scattering parameters of the device with the connectors and tapering in air, with and without the empty PDMS reservoir, are presented in Fig. 2a,b (black and light gray lines). In the same figure, measured curves for the entire GCCPW (including tapering and connectors) in air with and without the empty PDMS reservoir are presented (blue and light blue lines identified with the term “connector”). Finally, measurements were also performed on the non-connected device using the probe station, as described in the Materials and Methods, for the GCCPW in air with and without the PDMS reservoir. Since, in this last case, the measurement planes did not include the connectors and tapering of the GCCPW, these set of data are indicated as “without connector” and reported for the sake of clarity in the Supplementary Material associated to this paper.

S_11_ under these conditions (Fig. 2a) are, on the whole analyzed band, below −15 dB demonstrating a very good adaptation of the GCCPW and hence the suitability of the performed analytical design and numerical optimization. A rather good agreement between simulations and measurements was attained for the S_11_ curves. The presence of the connectors for the GCCPW in air does not essentially alter the device behavior in terms of return loss (S_11_) in comparison with the non-connected system (see Supplementary Material).

Simulated S_21_ (Fig. 2b) are in rather good agreement with the measured curves in the presence of connectors. A S_21_ of about −3 dB is shown at 3 GHz. These losses are related not only to spurious signal degradation introduced by the connectors but also to a resistive behavior presented by the deposited metal showing a resistance of nearly 5 Ω along the central conductor length of 56 mm due to its nano-metric thickness (about 170 nm).

The emission of the GCCPW in air was studied using numerical simulations. This aspect is important to evaluate the device radiation. In Fig. 2c the power density emission at different distances above and below the GCCPW substrate plane is presented for an input voltage of 1 V at the center of the device. The emission above the GCCPW is stronger than the one computed below the device substrate. This effect is due to the presence of the ground plane and the ITO layer that clearly limit the radiated electric field levels. At longer distances, the discrepancies among the emissions above and below the GCCPW tend to decrease due to the field lines coupling with the ground electrodes of the device.

### Loaded GCCPW analysis in the frequency domain: S parameters, emitted power densities and dosimetry

To validate the device design and fabrication modalities, it was necessary to proceed with the characterization of the GCCPW performances in the presence of the biological sample.

**Figure 3:**
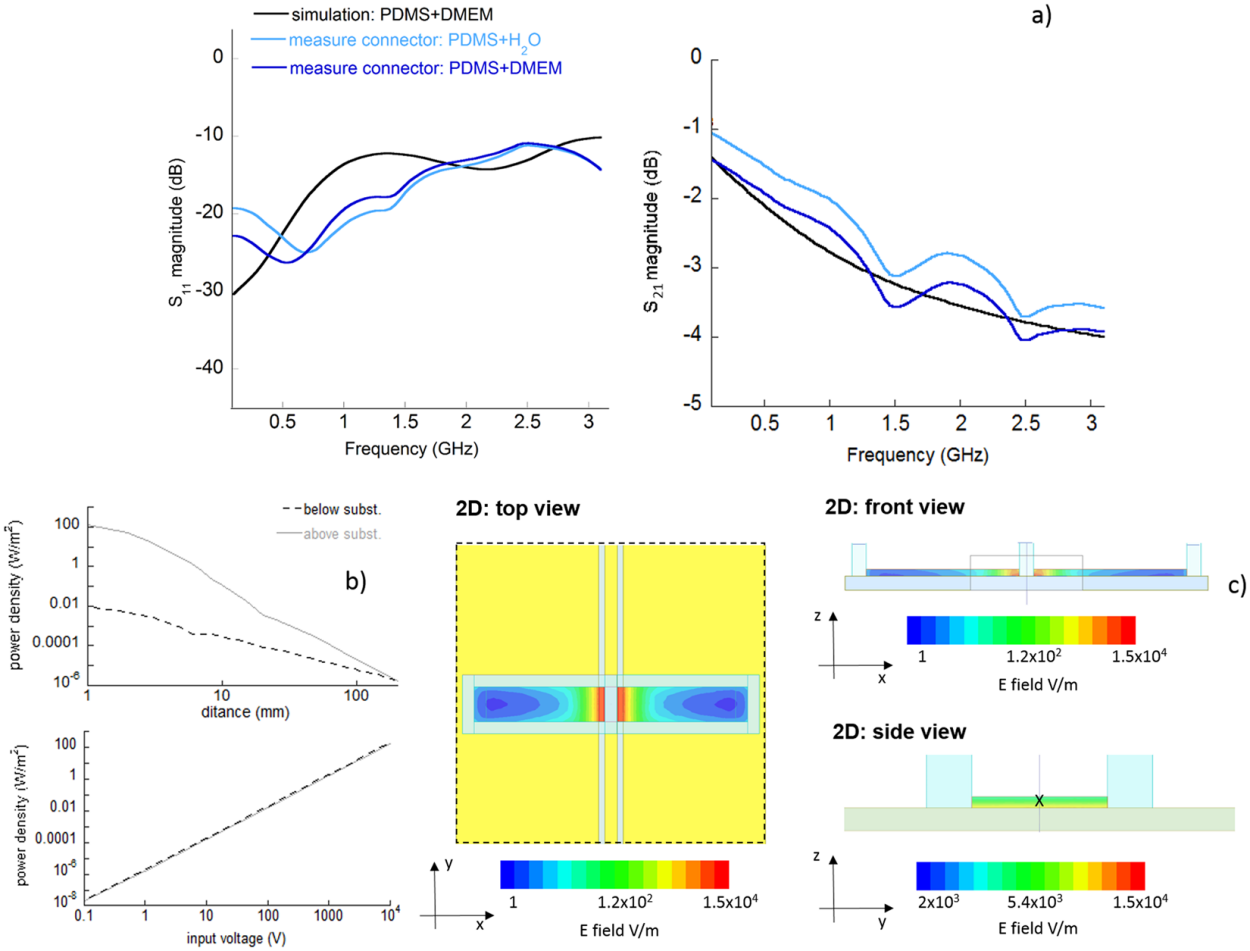
(**a**) The simulated S_11_ and S_21_ and the measured ones of the GCCPW with connectors and with the PDMS reservoir filled with an optimized volume of distilled water or DMEM. (**b**) Simulated emitted power densities at different distances for an input voltage of 1 V (top panel) and the emitted power densities at 200 mm apart from the GCCPW plane as a function of the input voltages (bottom panel). (**c**) Top (x-y), front (x-z) and side (y-z) views of the electric field spatial distribution in the DMEM solution at 1 GHz.

The measured scattering parameters of the connected GCCPW with the PDMS reservoir filled with a non-conductive (distilled water) and a conductive buffer (Dulbecco’s Modified Eagle Medium - DMEM) are presented in Fig. 3a. DMEM represents the matching worst-case condition. S_11_ parameters increased in the presence of the biological solution and water with respect to the GCCPW in air (Fig. 2a). However, they always remained below −10 dB up to 3 GHz. As expected, simulations and performed measurements are in good agreement. Globally, these data demonstrate the appropriate matching of the structure coupled with the biological media up to at least 3 GHz.

The S_21_ parameters are in line with the ones obtained for the device in air (see Fig. 2). With the connectors and the tapering, a slight reduction of the measured S_21_ is observed when the PDMS reservoir is filled with the conductive buffer (blue line) due to ionic losses. Measurements of scattering parameters for the non-connected GCCPW are reported in the Supplementary Material to this paper.

The presented results, that are the S parameters of the loaded device, were obtained after numerical optimization of the volume of the biological solution and of the dimensions of the ground aperture. These two points are important in order to have a good tradeoff between matching behavior, suitable amount of the biological solution to be exposed, and emission reduction. Simulations were performed varying l (see Fig. 1) between 3 mm and 9 mm and L (Fig. 1) between 1 and 22 mm at steps of 1 mm. The height of the sample holder was varied between 0.5 and 4 mm. In these simulations the volume of biological solution varied between 10 and 100 µL. As a result of this set of simulations, the optimum amount of liquid to minimize mismatches was found to be in the order of 30 µL for each rectangular holder (as those used in our measurements); hence the PDMS reservoir optimized dimensions are l = 3 mm and L = 20 mm for a height of 1 mm. The corresponding square ground aperture has an optimized side of 3 mm. A summary Table for these data is reported in the Supplementary Material.

The emission of the GCCPW with the PDMS reservoir filled with DMEM was studied using numerical simulations. This aspect is important to evaluate the safety operational regions for instruments that should operate in proximity of the GCCPW. In Fig. 3b, the power density emission at different distances above and below the GCCPW substrate plane is presented for an input voltage of 1 V. In direct proximity of the GCCPW the above emission is stronger than the one computed below, due to the presence of the ground plane and ITO that limits the electric field levels. At longer distances, the emissions above and below the GCCPW become equivalent, due to the coupling of the lateral field lines and their closing towards the ground plane of the device. The IEC 61010-1 standard that regulates the electric hazards for microscope producers in Europe specifies that radiated power density should not overcome 10 W/m^2^ at less than 50 mm apart from any electronic part included in the microscope. At a distance of 200 mm above and below the microscope stage the power density was evaluated for different input voltages and the limit for radiation safety was found for an input voltage higher than few kV. This result validates the safe use of the GCCPW integrated in a microscope stage under operation.

The representative distance of 200 mm was chosen as the head of the microscope, where the electronics are placed, is at 200 mm from the microscope stage.

These results were reported for the case of the PDMS reservoir filled with DMEM solution, as it is one of the most used culture media for cells. However, simulations for pure water were performed showing comparable data (not shown).

Finally, the electric field spatial distributions at different frequencies have been computed using numerical simulations. The electric field distributions at 1 GHz are reported in Fig. 3c, on a top, side, and lateral views. The maximum electric field magnitude reached within the exposure gap is above 14000 V/m for an input voltage of 7 V (i.e. 1 W on a 50 ohm load as given by the electromagnetic simulator), with an efficiency factor defined as the output (port 2) to the input (port 1) power ratio of about 1. Statistics of the electric field into the operational volume as defined in the Materials and Method are reported in Table 1. The coefficient of variation (CV) was computed and is reported into the same Table 1. At a height of 25 µm from the substrate surface, inhomogeneity of the electric field into the visibility windows (ROI in Fig. 1b) is of the order of 25%, which is in line with that of the exposure systems for *in vitro* bioelectromagnetic studies^55^. Inhomogeneity falls down to around 10% at a height of 10 µm from the substrate surface where usually cells are exposed and imaged. A similar inhomogeneity was found in both the right and left visibility window channels of the GCCPW (Fig. 1a) confirming the symmetry of the structure and hence the possibility to simultaneously expose two different samples (data not shown).

**Table 1:** Percentage of temporal delay and attenuation of transmitted ramps with rise times of 100 and 200 ps. Electric field statistics in DMEM solution in the frequency domain.

**Time domain analysis: temporal delays & attenuation**				
	*% rise time increase*		*% attenuation*	
	200 *ps*	100 *ps*	200 *ps*	100 *ps*
Without reservoir (air)	27	66	15	14
Empty reservoir	27	93	16	15
Reservoir non conductive	30	111	15	16
Drop of H2O	58	190	30	34
Reservoir conductive	38	112	20	21
DMEM drop	72	202	15	14
**Frequency domain analysis: E field statistics**				
***Frequency (MHz)***	***% CV of ROI solution height 25 µm***		***% CV of ROI solution height 10*** ***µm***	
100	28		11.2	
200	26.4		10.6	
800	27.8		11.12	
1000	25.3		10.12	
1600	23		9.2	
3000	25.5		10.2	

A similar inhomogeneity is reported in^56^, which deals with a bio-chip for electroporation applications, even if a direct comparison is not easily possible since in^56^ inhomogeneity was assessed in the time domain.

### Loaded GCCPW time analysis: reflectometry and electric field time trends

Time domain reflectometry data were acquired and different configurations of the GCCPW were tested considering either a drop of distilled water and DMEM just over the visibility windows, or the GCCPW with and without the PDMS reservoir filled with distilled water and the DMEM solution. Distilled water was tested because cells can be suspended and treated either in conductive culture buffer (DMEM) or in very low conductive media depending on the different applications and the specific biological observables.

**Figure 4:**
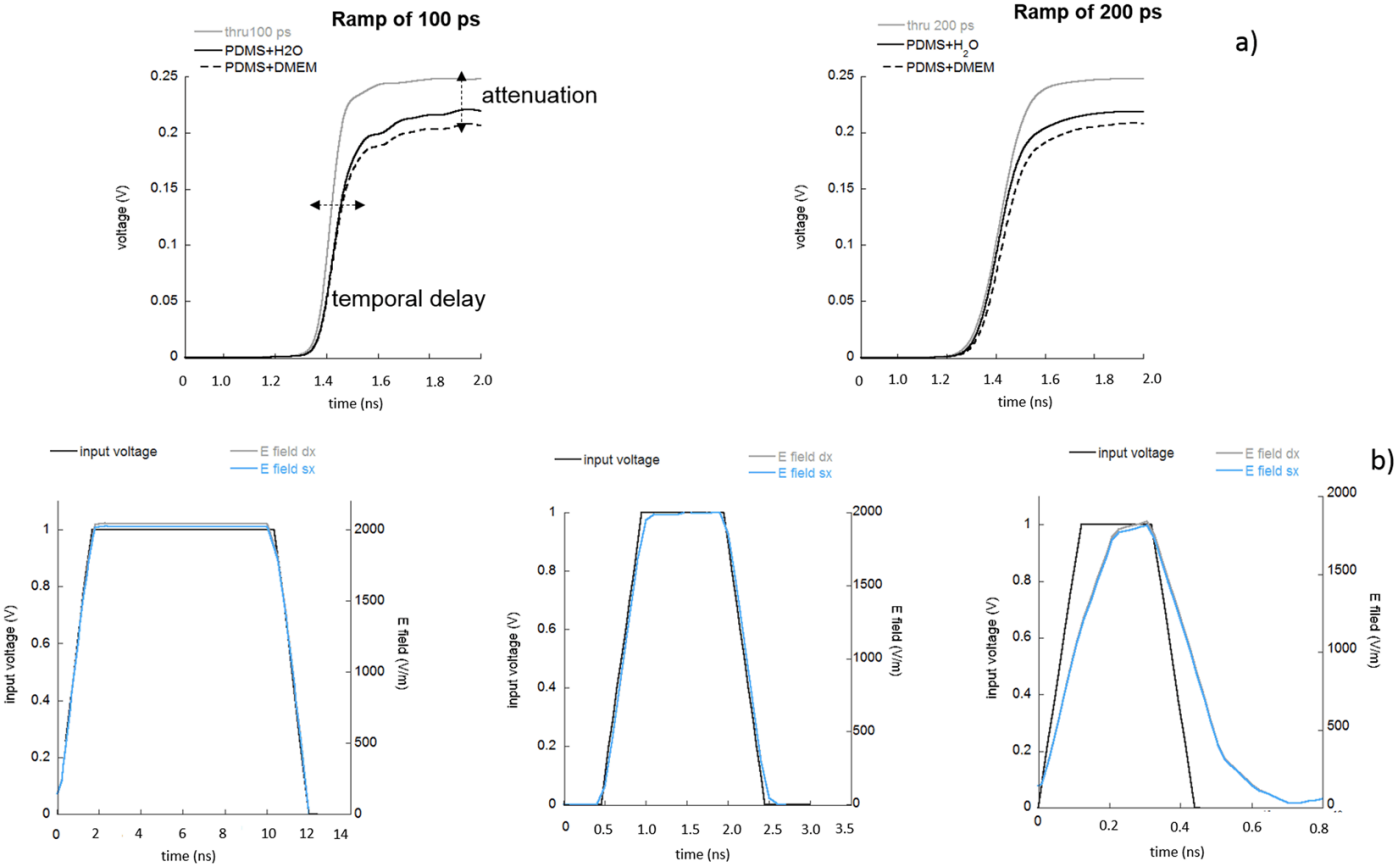
(**a**) transmitted voltage (ramp of 100 ps) through the “thru” (solid gray line) and the GCCPW prototype (left panel). Transmitted voltage (ramp of 200 ps) through the “thru” (solid gray line) and the GCCPW prototype (right panel). (**b**) Simulated time profiles of the electric field within 30 µL of DMEM in the point × sketched in Fig. 3.

Table 1 reports the increase in rise-times for two input voltage ramps, with respect to the ideal connection realized using a coaxial cable (named “thru”). The temporal delay is strongly reduced when the PDMS reservoir is used with either water or DMEM in comparison with the delay when a drop is placed over the GCCPW electrodes. Transmitted curves are presented in Fig. 4a, further showing a slight degradation of the device performance when the conductive solution is used.

Attenuation values under these conditions are reported in Table 1. Attenuations are slightly reduced when the PDMS reservoir is used, in lieu of the drop, especially when the conductive buffer is employed. This is due to the higher ionic content and dielectric associated losses. However, this decrease is rather low in all the tested conditions for the filled PDMS reservoir, and it never exceeds 20%.

Time domain simulations for electric field waveforms were finally performed using unipolar trapezoidal pulses of variable durations and rise/fall times. Specifically, pulses of 10, 1 and 0.4 ns were simulated with rise/fall times of 1, 0.5 and 0.1 ns respectively. In Fig. 4b, time trends of the electric field level in the centers of both visibility windows (see Fig. 1a,b for the visibility windows details) demonstrate the matching of the structure and the preservation of the electric field shape in the biological medium, in which the cells are placed during the experiments. Distortions and delays of the electric field waveform were introduced only when an input pulse of 400 ps was applied to the GCCPW. This result is in agreement with previous experimental evaluations performed using the time domain reflectometry analysis, which showed a substantial increase in time delays (Table 1) for the input ramp of 100 ps.

### CCPW biocompatibility

**Figure 5:**
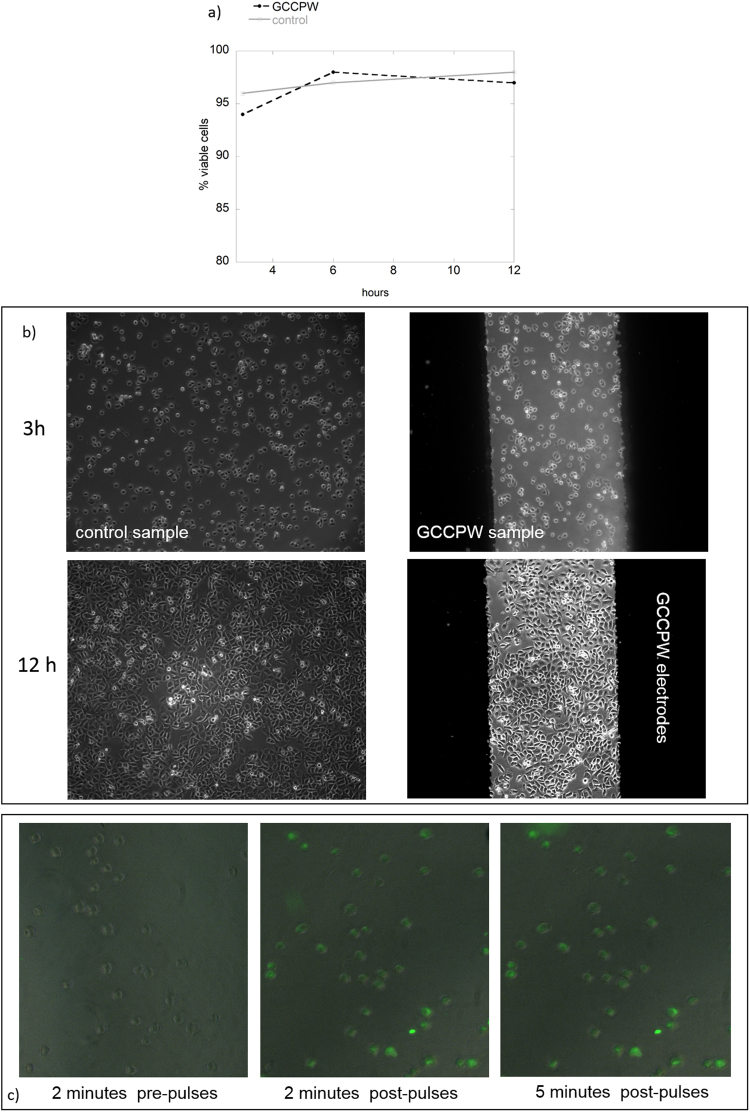
(**a**) Cell viability (average over three experiments) after 3, 6, and 12 hours of plating on a standard Petri dish or over the GCCPW electrodes. (**b**) Phase contrast images of the DC-3F cells after 3 and 12 hours of culture on a standard Petri dish or over the GCCPW electrodes. (**c**) Fluorescence images of the permeabilized haMSC cells.

The biocompatibility of the device was evaluated. Cell vitality was computed comparing cell growth on a standard petri dish and over the GCCPW electrodes. Cell vitality was not affected by the electrodes and the presence of the PDMS reservoir as evidenced in Fig. 5a. Each experiment was performed in triplicate. Contrast phase images at different time points (3, 6, and 12 h) were taken both for the control condition and for the exposed sample over the GCCPW electrodes (Fig. 5b), showing that cells growth and morphology were not affected.

An example of fluorescent images of haMSC cells before and after the pulses application is presented in Fig. 5c. Cells were pulsed in the presence of Yo-Pro-1 and the presence of fluorescent cells 2 and 5 minutes after the pulses delivery demonstrated the effectiveness of the GCCPW in cell permeabilization and direct imaging under the microscope.

It is worth to notice that images were acquired in the central area of the visibility windows (i.e. the ROI of Fig. 1b) further demonstrating the suitable electric field homogeneity reached as the majority of the haMSC cells were permeabilized.

## Discussion and Conclusions

In the literature various bio-chips for cell exposure have been already developed but their performances are not compliant with the requirements needed for real-time operation in bioelectromagnetics. One of the first limitations encountered in previous developed bio-chips is related to their frequency matching band that does not overcome 300 MHz^57–60^.

This limitation is not compatible with the need to expose cells to electric pulses with rise-times down to hundreds of ps or to microwave electromagnetic fields. Micro-devices specifically oriented to the dielectric spectroscopy of a single cell present wider frequency bands^61–63^ but are not suitable to be used with intense electromagnetic radiations. These devices are based on open coplanar waveguides with only small lateral ground electrodes. Our choice of a metallic enclosed electromagnetic bio-chip guarantees its matching on a wide band (from DC to few GHz), as it allows the propagation of the fundamental Transverse Electromagnetic mode (quasi-TEM) with a zero cutoff frequency and, at the same time, the drastic reduction of electromagnetic emissions. Concerning the matching bandwidth for high voltage devices, the best performance so far in terms of reflection coefficient is provided by^64^, where the S_11_ is below −10 dB up to 300 MHz. At 300 MHz the S_11_ of our GCCPW with and without the PDMS reservoir filled with DMEM medium is −22 dB, which is a dramatic matching improvement. Besides this good matching in reflection, our GCCPW presents an S_21_ always above −3 dB, up to 3 GHz. Therefore losses in transmission seem acceptable and in line with other devices for single or multicellular exposure as noted in^55,65^. Furthermore, reflectometry analysis was used for the first time to completely and accurately characterize the transmission properties of our GCCPW.

We assessed in detail the emission of the GCCPW. This is an important point in order to control the electromagnetic compatibility issues, which can arise especially when working in real-time with high voltages, as in the case of cells electropermeabilization under a microscope using nanosecond electric pulses. Our GCCPW ensures very low radiated power density of less than 1∙10^−6^ W/m^2^ (at 1 GHz) for 1 W applied, with respect to other similar planar structures (e.g. coplanar wave guides, microstirp lines, slot lines) due to the innovative design of the ground plane connection with the lateral coplanar waveguide electrodes.

The efficiency (ratio of the output to the input power) of our GCCPW is about 1, which is coherent with the negligible attenuation observed for the simulated electric field time trends, and which is one of the highest obtained so far. When 1 V is applied as input, the electric field is homogenously distributed with values in line with current literature^56,65^ and reaches around 2000 V/m in both exposure channels. This means that few kilo-volts are needed to deliver, over a large exposure gap (i.e. 500 µm), electric field intensities of some MV/m typical of the nanosecond electric pulses applications. On the other hand, just few hundreds of volts are needed for microsecond electropermeabilization. The bio-chip can therefore be used with already existing equipment and does not require unusual pulse generators.

The determination of the device transmission behavior, device emission and efficiency, incidentally, are disregarded or only poorly addressed in the current papers dealing with the similar subject^58,61,64,66^.

Our device is also fully biocompatible allowing cell growth and viability for at least 12 hours after cell plating over the GCCPW visibility windows and gold electrodes. The real-time monitoring of the cells is possible using inverted microscopes thanks to the use of the transparent conductive ground (ITO) covering the aperture on the ground plane, enabling also the possibility of detailed microdosimetric assessments of individual cells in their actual exposure conditions^67,68^. Suitability tests of the device for cell electropermeabilization and imaging using the fluorescence Yo-Pro-1 dye were performed.

To summarize, we provided a clear procedure for an optimized design of a biochip for bioelectromagnetic experiments. Such procedure involves as first step the device dimensioning based on analytical formulations, then its optimization via numerical simulations, its fabrication and experimental characterization based on frequency and time domain analyses. This systematic design, implementation, and validation of our GCCPW ensure a very good reproducibility and repeatability of the biological results.

All the imposed requirements for “second generation” experiments in bioelectromagnetics are satisfied by our new designed GCCPW. They deal with:

- a wide band system,
- the possibility of focusing laser beams of various diameters up to hundreds of µm in a transparent area,
- reduced setup emissions,
- high electric field homogeneity,
- a very good efficiency
- the biochip biocompatibility and its operational efficacy.

Therefore, our original GCCPW permits real-time and safe exposure of suspended or adherent cells for the visualization of the electromagnetic effects using in real-time various optical techniques both linear and non-linear.

## Materials and Methods

### Numerical simulations: GCCPW in air

We used numerical simulations (HFSS v. 15) to optimize the GCCPW dimensioning performed on analytical bases and to design the transitions toward standard connectors needed to connect the GCCPW to signal generators.

The simulated GCCPW included the presence of SMA connectors using standard dimensions for 50 Ω matching. Stimulation ports were placed on the concentric Teflon region of the coaxial connectors following the HFSS guideline instructions^69^; this step is critical to get correct simulation results. Metallization was made of gold with conductivity of 41 × 10^6^ S/m, while ITO conductivity was 5 × 10^5^ S/m.

Simulations in the frequency domain were carried out between 100 MHz and 3 GHz for an incident power of 1 W at the stimulating ports (a voltage of 7 V over the 50 Ω load represented by the bio-chip under investigation).

A cubic air box was considered around the GCCPW to account for the power density computation. The side of the box (750 mm) was equal to a quarter of the wavelength corresponding to the minimum frequency employed in simulations. An adaptive mesh of the simulation domain was used with a maximal dimension of 0.2 mm for the substrate and 2.5 mm for the air box. Simulations in air allowed defining the reflection coefficient (S_11_) indicating the amount of power reflected back by the bio-chip and the transmission coefficient (S_21_) representing the amount of power transmitted through the GCCPW. This latter parameter is important as it indicates the ability of the GCCPW to transmit the signal while avoiding its dissipation.

### Dosimetry

Simulations were also carried out loading the GCCPW with biological solutions. To keep cells in an appropriate medium, a suitable reservoir was conceived and its dimensions optimized using numerical analysis.

The double reservoir of PDMS (Fig. 1c) was simulated with an ε_r_ = 6.7.

The volume of the solution contained within the PDMS reservoir was varied changing the longitudinal length (l) of the holder as well as its transversal width (L), Fig. 1c. This strategy allowed us to define the best trade-off in terms of sufficient solution volume to assure cell vitality during experiments while maintaining the bio-chip adaptation.

Furthermore, the dimension of the square aperture within the ground plane were changed in accordance to the longitudinal length of the PDMS holder (l) in order to minimize radiations in the presence of the biological sample.

The biological solution was simulated as standard culture medium DMEM with ε_r_ = 76 and σ = 1.5 S/m. This latter value was measured using a conductimeter (Mettler Toledo LE703) while permittivity was as in^53^ for a similar buffer.

Analyses were conducted in the frequency domain and the electric field spatial distributions evaluated within the biological media. Statistical analyses of the distributions were carried out in the central volume of the visibility windows (ROI in Fig. 1b) at 0.15 mm apart from the central and the lateral electrodes, corresponding to the center of the visibility window where the exposed sample can be imaged during optical detection. It is worth noting that since imaging is possible focusing the light on single focal planes, the heights of the analyzed area were 25 µm and 10 µm. In the ROIs, the variation coefficients (CV), defined as the ratio between standard deviations and mean field values, were evaluated. CV indicates the electric field inhomogeneity within these volumes.

Power density emissions in the presence of the biological sample were also computed in order to respect the IEC Standards (i.e. IEC 61010-1) assuring a power density of less than 10 W/m^2^ for radiations between 100 MHz and 100 GHz within 50 mm from the microscope itself. Time domain simulations of the GCCPW using pulses of different durations were also carried out.

### Fabrication of the GCCPW

A prototype of the GCCPW was fabricated using photolithographic methods. Gold was deposed over a thin film of chrome and the ITO layer to fabricate (first) the ground plane after photolithography was performed using the S1805 resist. Then, gold was deposited on the other face after chrome sputtering and the electrodes were realized using a similar photolithographic process. To laterally close the device, gold depositions were realized on both GCCPW sides at the end of the two photolithographic processes. The device was connected (SMA connectors) using a conductive resin (no metallic soldering is possible on evaporated metallization) then silicone was added at the connector site to increase the isolation and mechanical stability.

The connected setup was then soldered on a PCB in order to create a solid substrate to give mechanical stability to the connector and to avoid the direct contact of the hands with the gold deposit where cells have to be placed. Final arrangement of the GCCPW is presented in Fig. 1d.

### Measurement of scattering parameters

Scattering parameter (S_11_) measurements of the fabricated GCCPW prototype were performed using a vector network analyzer (VNA, Agilent PNA-LN5230A) between 100 MHz and 3.5 GHz. A low phase variation coaxial cable was used to connect the device with the VNA. At the cable end the VNA was calibrated using a full two-port method with an electronic calibration kit (Agilent N469160006). Measurements were carried out with an IF bandwidth of 1 kHz over 3200 acquired points. Measurements were averaged over 10 acquisitions.

To experimentally assess the performances of linear tapering and connectors we also measured the S parameters using a coaxial probe station (Anritsu 37397C coupled to a CASCADE M150 system) directly on the bio-chip electrodes. Measurement planes, for this setup, are shown in Fig. 1d as dashed black lines.

Measurements of the GCCPW coupled with the PDMS reservoir filled with the solutions were always performed using a total volume of 30 µL in each container. The uncertainty of the performed measurements was largely within 1% as noted in^70^.

### Time domain measurements: transmitted signals

An oscilloscope equipped with the time domain reflectometry module (SDA 100 G Le Croy) was used for such measurements. The GCCPW was connected in a two-port configuration. At the port one, voltage ramp signals with rise times of 100 and 200 ps respectively were applied and transmitted signals trough the GCCPW were acquired at the port two.

Delays in rise times of the transmitted pulses were measured as well as the attenuation in the transmitted voltage to quantify signal distortion and losses through the fabricated GCCPW. Three measurements were performed under each of the following conditions: (i) device in air with and without the PDMS reservoir, (ii) device without reservoir with a drop of DMEM and distilled water (milli Q), (iii) device with reservoir filled with DMEM and distilled water.

Solution volume used during measurement was equal to 30 µl as in previous S parameters measurements.

Measurement uncertainty according to the instrument data sheet (type B) always remained within 10%.

### GCCPW biocompatibility and detection of cell permeabilization

To assess cell viability growing on the GCCPW, the trypan blue exclusion test was performed. DC-3F (Chinese Hamster lung fibroblast line) cells were incubated at 37 °C at 5% CO_2_ in Minimum Essential Medium (MEM) supplemented with 10% fetal bovine serum and 1% of penicillin streptomycin (Life Technologies, Cergy-Pontoise, France) for 3, 6, and 12 hours. This cell type was used because of its rapid growth. Control samples were placed into a 10 cm petri dish where a comparable PDMS container was placed directly over the dish bottom. The GCCPW used to verify the biocompatibility was similarly placed into a 10 cm Petri dish and the PDMS reservoir positioned over the GCCPW.

After the required incubation times, cells of both PDMS reservoirs were trypsinized and suspended in 1 ml MEM buffer. 10 µL of cells were taken and added to 10 µL of trypan blue. Live cells were manually counted.

To verify cell confluence and morphology in the two conditions, contrast phase images were taken at various time intervals using a Zeiss (Observer Z1 Zeiss, Marly-le-Roi, France) microscope (exposure time of 48 ms using a 10× objective).

To test cell permeabilization using the fabricated GCCPW, Yo-Pro-1 dye was added to human mesenchymal stem cells (haMSC). This cell type was used as its permeabilization threshold is well known^19^. haMSCs were grown in a humidified atmosphere at 37 °C and 5% CO_2_ in DMEM supplemented with 10% fetal bovine serum and 1% of penicillin streptomycin (Life Technologies, Cergy-Pontoise, France). Electropermeabilized cells were observed under fluorescence microscopy using Yo-Pro-1 uptake (Life technologies, Yo-Pro-1 λ_emission_ = 510 nm).

Cells were trypsinized and then re-suspended in DMEM, and then Yo-Pro-1 was added 5 minutes before the acquisition of the first image at a final concentration of 3 µM. To test cell permeabilization we delivered a classical electroporation protocol consisting in 8 electric pulses of 100 µs duration, with field amplitude of 2000 V/cm at a repetition rate of 1 Hz.

Images were acquired with an inverted microscope (AxioVert 100, with an exposure time of 300 ms on a 20× objective). Images were acquired 2 minutes before the pulses delivery, then 2 and 5 minutes after the exposure. Three independent experiments were performed.

### Data availability

The datasets generated and analyzed during the current study are available from the corresponding author on reasonable request. A part of these data are included in this published article as Supplementary Information file.

## Electronic supplementary material

Supplementary Information
