# Supplementary material for: A wide-band bio-chip for real-time optical detection of bioelectromagnetic interactions with cells
Source: Sci Rep. 2018 Mar 22;8:5044. doi: 10.1038/s41598-018-23301-w (PMC5864909; doi:10.1038/s41598-018-23301-w)
Supplement: Supplementary file 1 — Supplementary Information [file 41598_2018_23301_MOESM1_ESM.pdf]

# A wide-band bio-chip for real-time optical detection of bioelectromagnetic interactions with cells

Caterina Merla[a,b], Micaela Liberti[c], Paolo Marracino[c], Adeline Muscat[a], Antoine Azan[a], Francesca Apollonio[c], Lluis M. Mir[a]

[a] CNRS UMR 8203, Laboratory of Vectorology and Anticancer Therapy, Gustave Roussy, Univ. Paris-Sud, Université Paris-Saclay, 114 rue E. Vaillant, 94805 Villejuif, France.
caterina.merla@enea.it, adeline.muscat@gustaveroussy.fr, antoine.azan@gustaveroussy.fr, luis.mir@cnrs.fr.

[b] National Italian Agency for New Technology Energy and Sustainable Economic Development (ENEA) Division of Health Protection Technologies, via Anguillarese 301, 00123 Rome, Italy.
caterina.merla@enea.it.

[c] "Sapienza" University of Rome, Department of Information Engineering Electronics and Telecommunications, via Eudossiana 18, 00184 Rome, Italy.
liberti@diet.uniroma1.it, marracino@diet.uniroma1.it, apollonio@diet.uniroma1.it.

**Corresponding author:**
Caterina Merla, Ph.D.
address: CNRS UMR 8203, Laboratory of Vectorology and Anticancer Therapy, Gustave Roussy, University Paris-Sud, Université Paris Saclay, 114 rue E. Vaillant, 94805 Villejuif, France.
National Italian Agency for New Technology Energy and Sustainable Economic Development (ENEA) Division of Health Protection Technologies, via Anguillarese 301, 00123 Rome, Italy.
e-mail: caterina.merla@enea.it.
phone: +33 0142115091, +39 0630484616.

**Supplementary Material:**

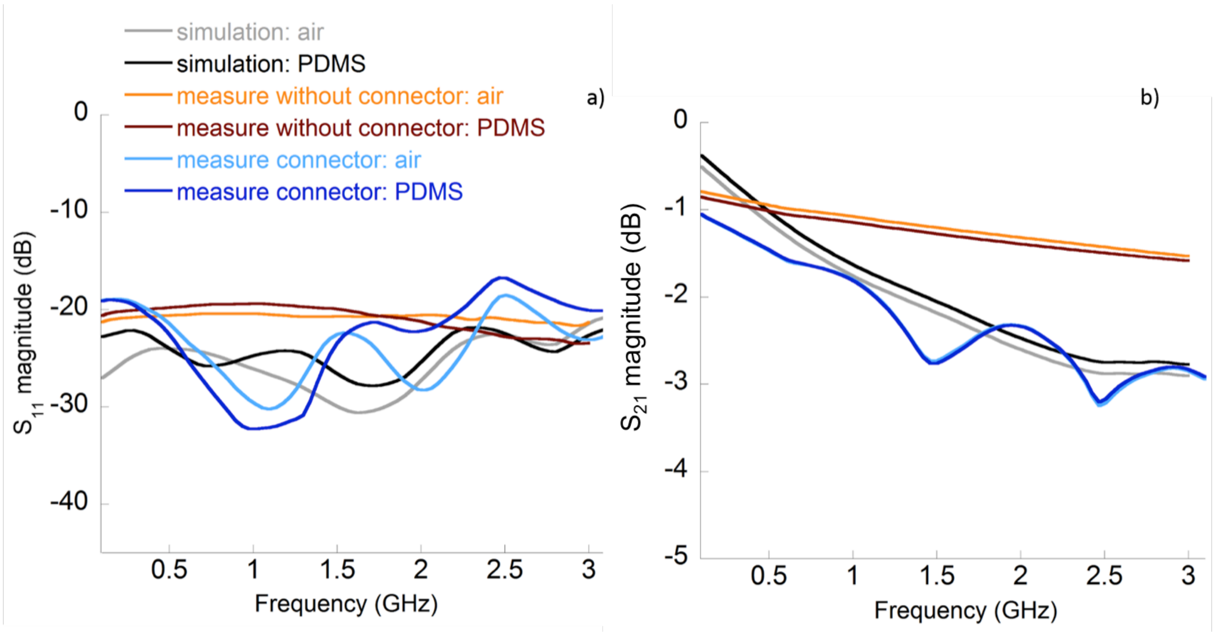


**Figure 1:** Scattering parameters of the GCCPW are presented in following conditions: i) simulations with and without the PDMS reservoir (gray and black solid lines) and ii) measurements with and without tapering and connectors with and without the PDMS reservoir (orange, purple, blue and light blue lines) respectively. $S_{11}$ are, on the whole analyzed band, below -15 dB (panel a). This result demonstrates a very good adaptation of the GCCPW. A rather good agreement between simulations and measurements is also attained for the $S_{11}$ curves. The presence of the connectors for the GCCPW in air does not essentially alter the device behavior in terms of return loss ($S_{11}$) in comparison with the non-connected device. $S_{12}$ parameters of the connected GCCPW (panel b) display increased losses compared to the non-connected device (orange and purple lines) due to the increased line length.

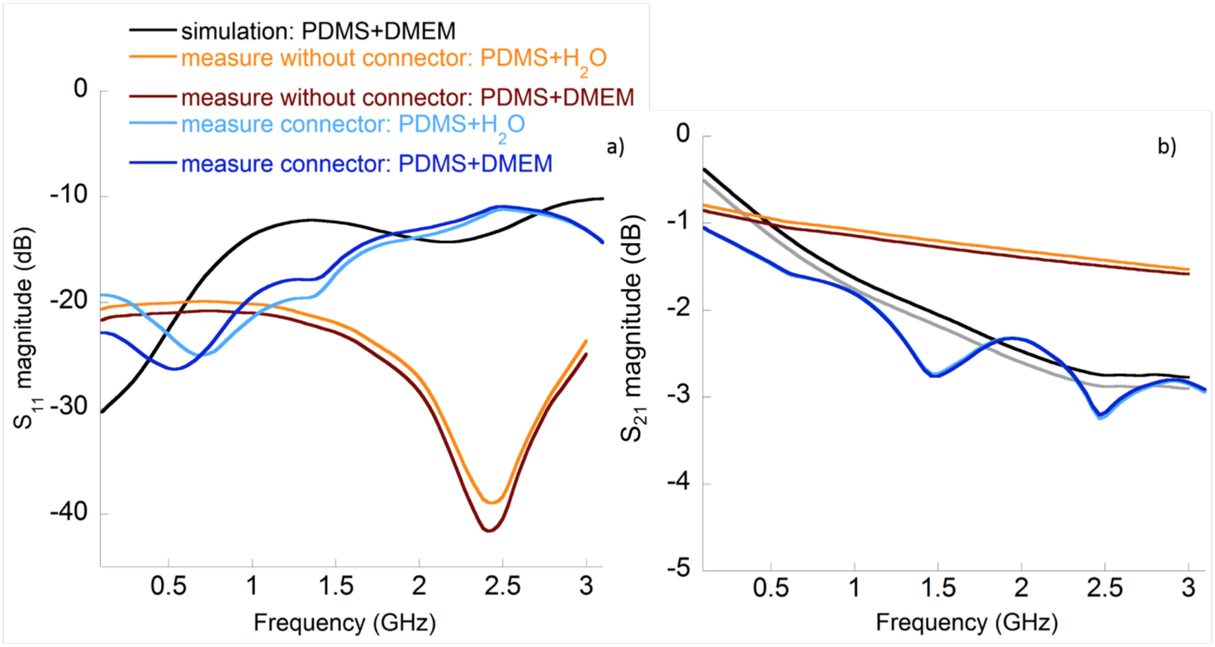


**Figure 2:** Scattering parameters (panel a and b) of the GCCPW are presented in following conditions: i) simulations with the PDMS reservoir filled with DMEM (black solid line) and ii) measurements with and without tapering and connectors with the PDMS reservoir filled with distilled water and DMEM (orange, purple, blue and light blue lines) respectively. $S_{11}$ are, on the whole analyzed band, below -10 dB (panle a). For $S_{11}$ and $S_{12}$ (panels a and b), the connected GCCPW (blue and light blue lines) shows a worst behavior with respect to the non-connected GCCPW (orange and purple lines) evidencing the role of the connectors and tapering to the global matching of the device.

Table 1:

| **Reservoir longitudinal length (l in Fig. 1, mm) fixing L at 1 mm** | **Max $S_{11}$ (dB)** |
|---|---|
| 3 | -12.0 |
| 5 | -10.0 |
| 9 | -7.0 |
| **Reservoir transversal width (L in Fig. 1, mm) fixing l at 3 mm** | **Max $S_{11}$ (dB)** |
| 1 | -12.0 |
| 5 | -11.0 |
| 10 | -10.5 |
| 20 | - 10.0 |
| **DMEM height (mm) fixing L at 20 mm and l at 3 mm** | **Max $S_{11}$ (dB)** |
| 0.5 | -10.5 |
| 1 | -10.0 |
| 4 | -10.0 |

Table 1: Optimization of the reservoir dimensions performed trough numerical simulation; summary of the main results obtained in terms of maximum reached $S_{11}$ values fixing alternatively the holder dimensions as shown in Fig. 1 of the main paper.
